# Discovery of functional genomic motifs in viruses with ViReMa–a Virus Recombination Mapper–for analysis of next-generation sequencing data

**DOI:** 10.1093/nar/gkt916

**Published:** 2013-10-09

**Authors:** Andrew Routh, John E. Johnson

**Affiliations:** Department of Integrative Structural and Computational Biology, The Scripps Research Institute, La Jolla, CA 92037, USA

## Abstract

We developed an algorithm named ViReMa (Viral-Recombination-Mapper) to provide a versatile platform for rapid, sensitive and nucleotide-resolution detection of recombination junctions in viral genomes using next-generation sequencing data. Rather than mapping read segments of pre-defined lengths and positions, ViReMa dynamically generates moving read segments. ViReMa initially attempts to align the 5′ end of a read to the reference genome(s) with the Bowtie seed-based alignment. A new read segment is then made by either extracting any unaligned nucleotides at the 3′ end of the read or by trimming the first nucleotide from the read. This continues iteratively until all portions of the read are either mapped or trimmed. With multiple reference genomes, it is possible to detect virus-to-host or inter-virus recombination. ViReMa is also capable of detecting insertion and substitution events and multiple recombination junctions within a single read. By mapping the distribution of recombination events in the genome of flock house virus, we demonstrate that this information can be used to discover *de novo* functional motifs located in conserved regions of the viral genome.

## INTRODUCTION

Viruses are renowned for their ability to mutate and rapidly adapt to new environments. Recently, the use of next-generation sequencing (NGS) has risen dramatically in virus discovery and the identification of emerging pathogens (1–3), the characterization of the human virome (4), the analysis of established infectious agents (5,6), the quality control of live attenuated viruses (7,8) and to understand the mutant spectra of viruses. NGS can be used to discover and characterize both homologous (9) and non-homologous recombination (10). Viral recombination generates considerable genetic diversity and plays a central role in the evolution and emergence of new viruses (11). Recombination can reshuffle single mutations that originally occurred on different, but homologous, viral genomes, resulting in the accumulation of advantageous mutations or the removal of deleterious ones. Homologous recombination between co-infecting viruses may also result in the evolution of new virus strains, as was observed among picornaviruses including human rhinoviruses (12) and between vaccine-derived polioviruses and circulating enteroviruses (13).

Non-homologous recombination has the potential to mutate large swathes of the viral genome by deleting large portions to form ‘defective genomes’ or by inserting foreign genetic material from other viruses or from the host. Defective genomes evolve during persistent and acute infections in cell culture (14) as well as during wild infections (15,16). The evolution of defective genomes was proposed to be critical in the transition of acute to chronic viral infections (17) and was found in patients persistently infected with measles virus (18), dengue virus (19) and hepatitis C virus (20). Virus-to-host recombination events are relatively rare as compared with intra-viral genome recombinations; however, such events may have important biological consequences such as the selective advantage conferred to hepatitis E virus on insertion of a 171-nt fragment from human S17 ribosomal protein mRNA (21).

We have developed a new algorithm called ViReMa (**Vi**ral-**Re**combination-**Ma**pper) to provide a versatile platform for the discovery of recombination events in deep sequencing datasets. ViReMa is compatible across a number of sequencing platforms that produce either long (e.g. 454 technologies) or short (e.g. Illumina) reads and can work with a variety of viral genomes (DNA or RNA, single- or double-stranded, multi- or single partite, short or long, etc). ViReMa does not require any pre-treatment of the original dataset beyond standard quality-filtering and does not require any special reference library generation. In addition to single recombination events, multiple recombination events within a single read are detected, as are virus-to-host recombination and insertion and substitution events. Using ViReMa, we demonstrate that by mapping the distribution and frequency of recombination events in the genome of flock house virus (FHV), we can discover *de novo* functional genomic motifs required for viral replication and encapsidation. Source code and updates for ViReMa can be found at sourceforge.net/projects/virema

## MATERIALS AND METHODS

The dataset analyzed in this study was generated as part of a previous analysis in our laboratory (10) and is publicly available at the NCBI Small Read Archive with the accession number SRP013296. These reads are 100-nt single reads generated on an Illumina HiSeq 2000 using standard cDNA library generation protocols and directional RNAseq.

Briefly, authentic FHV particles were amplified in *Drosophila* S2 cells grown in suspension, harvested 2 days after infection and then purified over a series of centrifugation steps consisting of one 30% sucrose cushion and two 10–40% sucrose gradients in the presence of 50 mM Hepes, pH 7.0. An additional 2-h nuclease digestion was performed with 20 U DNase I and 0.5 ug RNase A in between the two sucrose gradient spins to remove any contaminating non-encapsidated RNA or DNA. After the final sucrose gradient, RNA was extracted using standard phenol/chloroform extraction, ethanol precipitated and re-suspended in pure water. In all, 400 ng RNA was used for cDNA library generation using standard TruSeq adaptors and barcodes, polymerase chain reaction amplified for 12 cycles and then purified by agarose gel electrophoresis to yield inserts of ∼200 nt. The cDNA library was loaded onto a HiSeq v3 single read flowcell and sequenced for 100 nt of the insert and 7 nt of the index sequence on an Illumina HiSeq 2000. Reads were processed using CASAVA 1.8.2.

Before analysis, the raw reads were processed by removing the 3′ adaptors (sequence = TGGAATTCTCGGGTGCCAAGG) using Cutadapt (22) with default parameters and then removing any reads containing any nucleotide with a PHRED score < 20 using the FASTX toolkit (http://hannonlab.cshl.edu/fastx_toolkit/). Any read <50 nt was discarded, leaving 31 146 304 reads ranging from 50 to 100 nt in length. For the analysis using pseudo-reference libraries to detect RNA recombination, the last five nucleotides of each read were trimmed away and then any read containing <95 nt was discarded. This yielded a dataset containing 28 939 991 reads, all exactly 95 nt in length. These datasets were then aligned end-to-end to the FHV genome (NC_004144 and NC_004146) using Bowtie (version 0.12.9) with parameters -v 2 –best, and then to the *Drosophila melanogaster* genome (fb5_22) using parameters -v 3 –best. Any unaligned reads were analyzed for evidence of recombination as described in the main text.

## RESULTS

**Figure 1.**
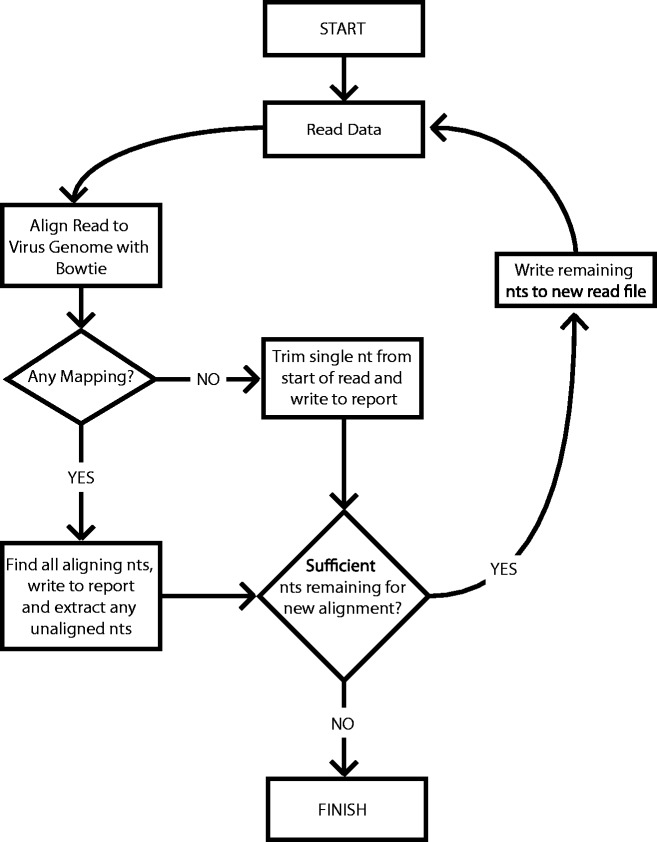
A flow diagram of the ViReMa algorithm. Input data can be either in FASTA or in FASTQ format and can contain reads of any length up to 1024 bp. ViReMa initially attempts to map the 5′ end of a read to the reference genome(s) using Bowtie. If a mapping is found, ViReMa generates a new read segment from any unaligned nucleotides at the 3′ end of the read. If not, ViReMa trims the first nucleotide. If there are sufficient nucleotides present in the new segment, it is written into a new data file and again mapped by Bowtie. This continues iteratively until all portions of the read are either mapped or trimmed or a combination of both.

**Figure 2.**
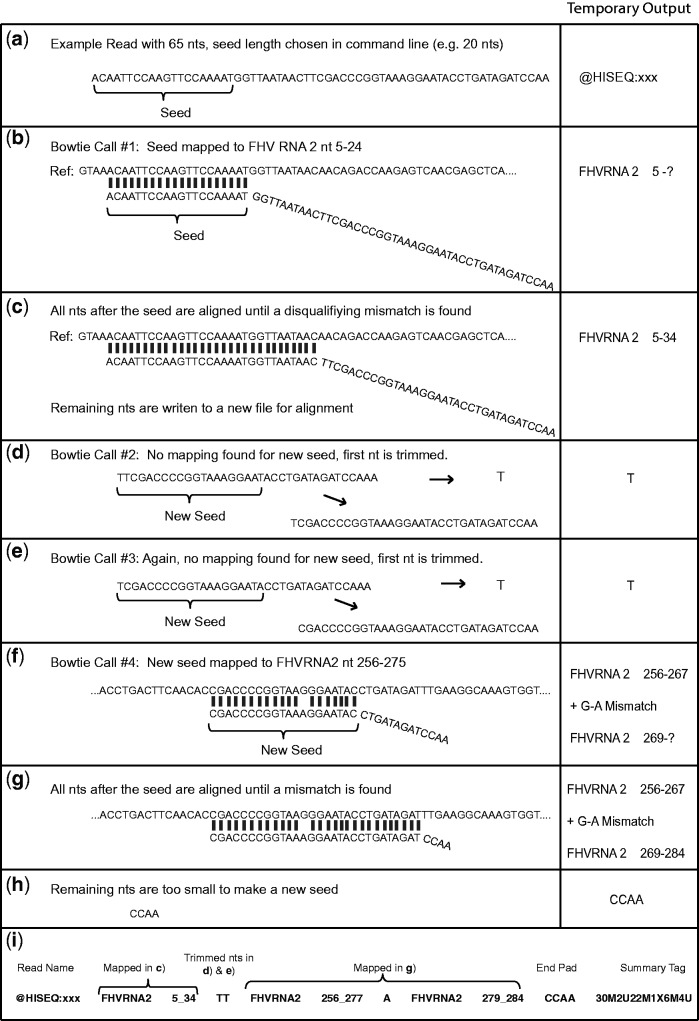
An example read is mapped with ViReMa. (**a**) A typical short read containing 65 nt. A seed length of 20 nt is chosen and is mapped to the FHV genome. (**b**) A good alignment is found from nt 5–24 in FHV RNA 2. (**c**) Ten further nucleotides are found after the seed that map at this location in the reference genome until a mismatch is found. The remaining nucleotides thus form a new segment. (**d**) A new seed is extracted from the new segment, but a successful mapping cannot be found. Consequently, the first nucleotide is trimmed, and again a new segment is generated. (**e**) For a second time, a mapping cannot be found for the seed and the first nucleotide is trimmed to form a third segment. (**f**) Finally, a mapping is found for the third segment at nt 256–275 on FHV RNA2 that contains one mismatch at nt 268. (**g**) Nine further nucleotides are found after the seed that map at this location in the reference genome until a mismatch is found. (**h**) The remaining nucleotides form a new segment, but it is shorter than the required seed length and so is not aligned to the reference genome. (**i**) Finally, the results of these mapping events are recorded and appended with a summary code.

ViReMa is a Python script that iteratively calls the small read alignment program, Bowtie (23), to try and map all portions of a candidate read. The process used is similar to other recombination or fusion detection algorithms such as Tophat and Tophat-Fusion (24,25), FusionMap (26) or MapSplice (27) that split up a read into segments of a specific length, which are then mapped independently. However, ViReMa initially attempts to align the 5′ end of a read to the reference genome(s) and then dynamically generates a new read segment by either extracting nucleotides at the 3′ end of the read that fail to align or by trimming the first nucleotide from the read. This continues iteratively until all portions of the read are either mapped or trimmed or a combination of both as summarized in the flow diagram in Figure 1. This process is illustrated step by step using an example read in Figure 2. ViReMa is split into two phases. The first phase searches and reports recombinations found for each read of a given dataset. The second phase compiles these results into several output files based on the recombination events that they describe.

### Mapping phase

ViReMa uses Bowtie’s seed-based alignment: ‘–n’ mode. A seed, typically comprising 20–30 nt, is extracted from the beginning of a sequence read and aligned to a reference genome using a Burrows–Wheeler indexing technique (23). If a valid mapping location is found for the seed, the remaining nucleotides are also aligned. Bowtie takes into account the quality scores for each nucleotide in the read and reports a successful mapping provided that the sum of the quality scores for each discovered mismatch does not exceed a user-defined value: the ‘-e’ value. If multiple mapping locations are found in the reference genome, Bowtie (in ‘best’ mode) reports the mapping that raises the fewest mismatches in and after the seed.

ViReMa exploits this method of mapping by purposely specifying a very high ‘-e’ value. Consequently, Bowtie will report the locations of a successfully mapping seed regardless of the number of mismatched nucleotides that follow it. These mismatches are reported in the 13th field of the standard sequence alignment map (SAM) output (28). For example, a 75-nt read that successfully aligns to a reference sequence but with only one mismatch might report ‘MD:Z:45C29’ in this field. However, a 75-nt read that maps over a recombination junction might read: ‘MD:Z:45C0G0G0C0C0G3G0A0G0G0A0T0C0C0A0A1T0T0T1A1G0T0C1’. From this, we can determine that the first 45 nucleotides from the read have been successfully aligned, but the remaining 30 nucleotides are predominantly mismatched to the reference sequence. (Note that the CIGAR string will still read ‘75M’.) The first 45 aligned nucleotides thus correspond to the 5′ region of a putative recombination junction up until the recombination breakpoint. The remaining nucleotides in the read after a detected breakpoint can then be extracted from the sequence alignment map file and used to generate a new read segment for use in another Bowtie alignment.

A new segment will be generated from the remaining nucleotides beginning with the first disqualifying mismatch. ViReMa can tolerate up to two mismatches (‘–N’ parameter in command line) in the seed during the Bowtie mapping and in the remaining aligned nucleotides. However, these mismatches cannot occur in the nucleotides immediately preceding or following a putative recombination event (‘–X’ parameter in command line). This ensures confidence in the discovery of a recombination junction while allowing maximum sensitivity of the segment mapping. This also prevents a segment from mapping beyond the true recombination junction by claiming mismatched nucleotides at this location. Not only would this introduce noise, this would reduce the number of nucleotides used in subsequent mapping iterations that can be mapped to the 3′ side of the recombination junction, which would therefore reduce overall sensitivity.

ViReMa also has the capacity to search a host genome for putative recombination events. ViReMa will first attempt to align the seed to the virus genome. If a mapping cannot be found, ViReMa will then attempt to map the read to the host genome. This will give preference to a mapping found in the virus genome, even if a better match may have been found in the host genome. Additionally, it is possible to specify different seed lengths when mapping to either the host or viral genome.

If a successful alignment is not found during the mapping of a segment, then the first nucleotide is trimmed and a new segment is formed from the remaining nucleotides. This trimming process is crucial to remove short pads or adaptor sequences from the beginning of a sequence read that might prevent a seed from being mapped. Trimming may also occur in the middle of a read after one or more segments have already been mapped. This enables ViReMa to read through insertion events or regions of extensive mismatching. The nature of these trimmed nucleotides is scrutinized in the second phase of the program and is reported according to whether they constitute insertion or substitution events. This process will also find regions where multiple recombinations have occurred, as discussed later, in Compound_Handling.

In a worst-case scenario, ViReMa will be unable to map any portion of a candidate read and so would trim off each nucleotide from the beginning of the read in multiple iterations until there are too few nucleotides remaining to form a segment long enough to be mapped. Consequently, a poor-quality dataset or an incomplete or inaccurate reference genome will result in a large number of Bowtie calls and a corresponding increase in run time.

Once an entire read has been mapped, or there are fewer nucleotides left in a segment than required by the seed length, the mapping results are written to an output file. Each entry is appended by a short code to summarize the results of the mapping: ‘M’ corresponds to mapped nucleotides; ‘X’ denotes mismatches within mapped segments; and ‘U’ denotes unmapped nucleotides that were either trimmed or could not form a new segment as required by the seed length. This code aids the second phase of the algorithm that analyzes the mapping results for each read and identifies what types of events have occurred. Examples of the types of output generated by the first phase of ViReMa are given in Table 1.

**Table 1.** Examples of output from mapping phase of ViReMa

a)	@Read:name FHVRNA1 113-145 FHVRNA1 1003-1045 **33M43M**
	A straight-forward recombination event
b)	@Read:name FHVRNA2 1000-1050 Chr3R 8273432-8273467 **51M36M**
	A virus to host recombination event
c)	@Read:name FHVRNA1 50-100 FHVRNA1 700-730 FHVRNA1 1140-1165 **51M31M26M**
	Two recombination events found within a single read
d)	@Read:name FHVRNA2 110-140 GGGCCCGAG FHVRNA2 141-175 **31M9U35M**
	An insertion of 9 nt
e)	@Read:name FHVRNA2 110-140 GGGCCCGAG FHVRNA2 150-184 **31M9U35M**
	A substitution of 9 nt
f)	@Read:name ACTGAC FHVRNA1 2500-2560 ACGCCGA **6U61M7U**
	A read with a single mapping, but with pads at either end
g)	@Read:name FHVRNA1 500-550 ACGCCGAACTACGACGACTATTCGATC **51M27U**
	Unknown or ambiguous recombination event: Initially mapped, but then pad is longer than seed (e.g. 25 nt) and would be long enough to BLAST
h)	@Read:name FHVRNA1 500-550 ACGCCGGGCAG FHVRNA1 1040-1080 **51M11U41M**
	Unknown recombination: recombination has taken place, but unidentified nucleotides are present that are smaller than the chosen seed (e.g. 25 nt). This may be the result of two recombination events having occurred within proximity, which can be tested using the optional command: ‘–Compound_Handling’
i)	@Read:name ATAGCATGCAGCGTTATTTAGCACGACAGAATCATCGACTAGCTACGAT **44U**
	An unmapped read

The output from the mapping phase of ViReMa gives the name of the read, followed by the details of each successfully mapped segment or the nucleotides that were trimmed, and are appended with a code to describe these mappings.

### Compiling and reporting results

In the simplest case, a recombination event is found when a read is mapped to two separate locations on the same gene and there are no other unmapped nucleotides present. Such an example is given in Table 1 (a). Here, a read that is 76 nt in length has been broken into two segments, each of which has been perfectly matched to the virus genome as denoted by its code ‘33M43M’. The first segment of 33 nt has been mapped to FHV RNA 1, nt 113–145, and all the remaining 43 nt have mapped to FHV RNA 1, nt 1003–1045. Therefore, this read maps across a single recombination event between nt 145 and 1003. In a similar manner, multiple or inter-species recombination events can also be detected [as in e.g. Table 1 (b or c)]. ViReMa is also sensitive to complex recombination events, insertions, substitutions and can also map reads that contain short pads at either or both of the 5′ and 3′ end of the read. Examples of these are given in Table 1.

The exact site of recombination is sometimes ambiguous due to the inherent ‘fuzziness’ of recombination junctions. This occurs when the nucleotides immediately upstream of the acceptor site are identical to the nucleotides immediately upstream of the donor site. This leaves a number of possible sites where the original recombination event may have occurred but would still produce an identical resulting sequence. In ViReMa, such ‘fuzzy’ junctions can be reported either at the 5′ end, the middle or at the 3′ end of the fuzzy nucleotides present in the reference sequence, as chosen by the user using the –Defuzz parameter.

Once each read has been scrutinized, each type of recombination event is tallied and these results are written to specific output files used for downstream analyses: Insertions, MicroInsertions, MicroDeletions, Recombination_Results, Single_Alignments, Substitutions, UnMappedReads and Unknown_Recombinations. If both host and virus reference genomes are used, there will be one of each file for both reference genomes, plus an extra file for virus-to-host recombinations.

### Compound handling

The accumulation of multiple recombinations within single template can result in a highly fragmented and complex recombinant genome. This raises problems when trying to identify the original individual recombination events that took place. The output of such a scenario may look like the example in Table 1 (h). Here, segments at the 5′ and the 3′ end of a complex recombination event have been mapped to nt 500–550 and nt 1040–1080 of FHV RNA 1, but there remain a small number of trimmed nucleotides in the middle.

During the second phase of ViReMa, the ‘–Compound_Handling’ option can be used to attempt to align these short fragments back to the virus genome. The two flanking aligned segments provide a small window in which to search for a new alignment. As this window would be considerably smaller than the entire viral genome, a reliable mapping for this fragment may therefore be found even if the fragment is smaller than the seed length. If a single perfect match is found, then ViReMa will report two separate recombination events as opposed to one ‘unknown’ recombination. For example, in the case of the example in Table 1 (h), an unknown sequence is flanked by two segments that map to nt 500–550 and nt 1040–1080 of FHV RNA 1. Using ‘–Compound_Handling’, this sequence is found to correspond to FHV RNA 1, nt 700–710 and so ViReMa will report this as two recombination events: one from FHV RNA 1, nt 550 to 700; and the other from FHV RNA 1, nt 710 to 1040.

### Analysis of recombination events using simulated data

To assess the sensitivity and error rate of ViReMa, we generated a simulated dataset containing randomized recombination events in FHV RNA 1. To generate a simulated read, a 94-nt fragment was randomly selected from FHV RNA 1 and then appended to another randomly selected 95-nt fragment. From this 189-nt fragment, a read of 95 nt was extracted starting from a randomly chosen nucleotide. In this manner, a recombination event could occur at any position along the synthetic read. For each 189-nt fragment, this was repeated a random number of up to 100 times. There are 94 possible ‘cutting’ sites in a 95-nt read at which a recombination may occur. With a search seed of 20 nt, recombination events occurring in the first or last 19 cutting sites of the reads will not be detected leaving 56 possible sites. Therefore, a theoretical maximum efficiency of recombination detection would be 56/94 = 59.6%.

We generated 5 043 791 synthetic reads containing 99 033 unique recombination events and aligned these reads to the FHV genome with ViReMa using a seed length of 20 nt. We detected 2 973 603 recombination events, which correspond to a detection sensitivity of 59.0%—just below the theoretical maximum. As we know the nature of the simulated recombination events, we can determine that no incorrect events were reported.

**Figure 3.**
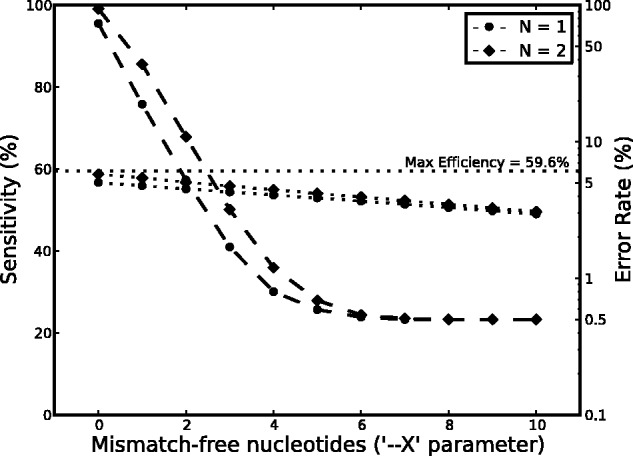
The sensitivity and error rate of ViReMa determined using a simulated dataset. A simulated dataset containing 5 042 282 reads was analyzed by ViReMa using either an allowed mismatch rate of N = 1 (circles) or N = 2 (diamonds) and an ‘–X’ value of between 0 and 10. The sensitivity (left axis, dotted line) decreases linearly with increasing mapping stringency as imposed by the ‘–X’ value. The error rate (right axis, dashed lines) decreases dramatically with increased mapping stringency.

To further test the sensitivity and error rate of ViReMa, we generated another simulated dataset containing 5 042 282 reads, but including a random mismatch rate of 7.5 nt per 10 000 wild-type nucleotides. This approximates the error frequency previously reported for RNAseq datasets of RNA viruses including FHV (5,6). We aligned these reads to the FHV genome allowing either one or two mismatches per read segment (‘–N’ parameter = 1 or 2). In addition, we varied the number of nucleotides at both the beginning and end of each read segment in which these mismatches were disallowed (the ‘–X’ parameter). As can be seen in Figure 3, the sensitivity of recombination detection is close to the theoretical maximum when mismatches are allowed to occur anywhere in the read segment. However, the error rate of recombination junction detection is poor due to the first one or two nucleotides upstream of a recombination junction being counted as mismatches but in the downstream segment. By increasing the stringency of the recombination mapping with the ‘–X’ parameter, the error rate improves dramatically, but with a small penalty in sensitivity. The improvement in error rate plateaus with an ‘–X’ value of 5 nt for –N = 1, and an ‘–X’ value of 6 nt for –N = 2 (Figure 3).

Although this analysis does not account for the many other sources of potential error in deep-sequencing datasets, it can act as a guide to optimize search parameters in subsequent analyses. For example, from 5 042 282 simulated reads, we detected 2 654 538 recombination events using a seed length of 20 nt, –N = 1 and –X = 5. This results in a sensitivity of 52.9%. From these reported events, 15 647 were inaccurate, thus giving an error rate of 0.59%. These errors are due to mismatches overlapping the ‘fuzzy’ region of a recombination junction (as described earlier) and so are inaccurate by only a small number of nucleotides as limited by the size of the ‘fuzzy’ region.

### Analysis of recombination events in the RNA encapsidated by FHV

We recently reported that by deep-sequencing the RNA encapsidated by FHV, it is possible to detect a plethora of recombination events within the viral genome with a frequency approaching that of mismatch mutation (10). In that study, we collected 28 939 991 single reads that were quality filtered and all trimmed to exactly 95 nt. Using an end-to-end alignment, we removed all reads that mapped to either the FHV or *D. melanogaster* genomes (Table 2), leaving a dataset containing 3 225 981 reads. Next, we generated a pseudo-library containing ∼20 million short reference sequences corresponding to all the possible recombination events that might occur within the FHV genome. By aligning the unmapped reads to this pseudo-library (allowing two mismatches per read), we identified 1 186 678 recombination events (excluding insertions and deletions smaller than 5 nt, ‘MicroInDels’, that were found to be artifacts). Using these results, we generated a second pseudo-library corresponding to recombination junctions that occurred within 55 nt of one another, enabling us to identify an additional 160 960 recombination events. In total, we detected 1 346 768 recombination events using the pseudo-libraries with end-to-end mapping (Table 2).

**Table 2.** Comparison of pseudo-library and ViReMa methods for detecting viral recombination events

Mapping result	Pseudo-library	ViReMa	ViReMa
	95-nt reads	95-nt reads	All reads
Total reads	28 939 991	28 939 991	31 146 304

FHV genome	24 532 314	26 172 003	27 300 855
*D. melanogaster*	1 081 696	1 095 098	1 207 843
FHV RNA1-RNA1	1 126 364	1 306 711	2 353 262
-MicroInDels ≤ 5 nt	41 565	66 731	63 857
FHV RNA2-RNA2	26 958	26 449	29 702
-MicroInDels ≤ 5 nt	6939	6659	7874
FHV RNA1-RNA2	11 068	10 545	13 274
FHV RNA2-RNA1	22 288	23 357	24 573
Other recombination events (see Table 3)	160 090	18 111	20 149
Unmapped reads	1 990 779	4801	10 435

Total recombination events	1 346 768	1 385 173	2 440 960

The results for the pseudo-library-based mapping were performed in Routh *et al.* (10) and are compared here against the number of recombination events found by ViReMa when using the same initial dataset containing only 95-nt long reads. As ViReMa does not require uniform read lengths, the raw dataset containing 31 million reads was also mapped with ViReMa.

To compare the sensitivity of ViReMa with this approach, we analyzed the same dataset (3 225 981 reads) using similar parameters (seed length of 20 nt for virus alignment and 25 nt for host alignment, 1 mismatch allowed per segment and an ‘–X’ value of 5). Using a standard Apple Intel workstation with 16 Gb RAM and 8 physical core processors, this analysis was performed in <20 min. This yielded 1 385 173 recombination events (Table 2), an improvement in sensitivity of ∼3%. This small improvement is despite the fact that ViReMa uses a stricter mapping procedure. When determining which reads had been successfully mapped with the ViReMa algorithm, but not by the pseudo-library alignment, we found that all of these reads contained multiple events within the read. This is why the majority of the extra events were detected in FHV RNA 1, which harbored the largest number of recombination events among the FHV genome and many of which occurred in proximity to one another. Furthermore, in addition to the improved sensitivity, we were also able to detect several other events, including intra-host and host-to-virus recombination events, insertion and substitution events, and some ‘unknown’ events that contain fragments of either viral or host RNA as well as a large number of trimmed nucleotides (Table 3).

**Table 3.** Other recombination events detected using ViReMa

Mapping result	ViReMa	ViReMa
	95-nt reads	All reads
Virus to host recombinations	10 791	12 186
Host recombinations (e.g. splice sites)	7305	7949
Host MicroInDels	3375	3663
Unknown or ambiguous recombinations	171 918	180 075
Insertions >5 nt		
Host	5	10
Virus	3	3
Substitution events >1 nt		
Host	1148	1246
Virus	539	469
Mixed sense virus recombinations	15	14

ViReMa can also detect virus to host recombination events as well as recombination events in the host genome by including a second reference genome during the mapping phase.

ViReMa allows the analysis of datasets containing reads of variable lengths. In our previous analysis, we had trimmed all of our reads to a uniform length as is required when using the pseudo-library approach. However, a large improvement in sensitivity was achieved by analyzing the raw dataset that contained 31.1 million reads ranging from 50 to 100 nt in length. With this, we found 2 440 960 recombination events (Table 3), a dramatic improvement over initial analysis. After this, <11 000 reads remained that were completely unmapped.

### *De novo* discovery of functional motifs in the genome of FHV

FHV readily produces defective genomes, even during limited passaging in cell culture. Defective genomes have lost their ability to independently encode functional viral proteins and are dependent on the wild-type ‘helper’ virus for replication and propagation. The study of defective genomes has been highly important in establishing the regions of viral genomes required for replication and encapsidation (29). Defective genomes that maintain the sequence information required for replication or encapsidation gain a strong selective advantage over those that cannot and so will be successfully propagated and thus highly represented in our dataset (30). Conversely, recombination events that remove functional motifs are negatively selected and so these events are seldom observed.

**Figure 4.**
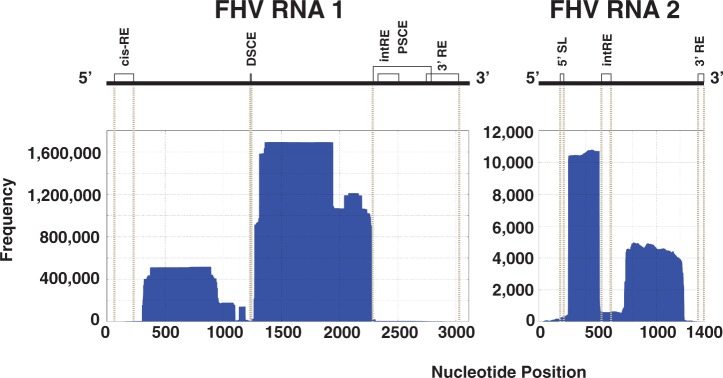
Survey of nucleotides in the FHV genome that are deleted after recombination. Schematic representation of RNA 1 and RNA 2 from the FHV genome indicating the nucleotide regions known to be required for either replication or encapsidation. The frequencies with which nucleotides were excised due to a deletion (blue) are plotted for FHV RNA 1 and FHV RNA 2. This reveals conserved regions of the FHV genome that are required for replication or encapsidation. In addition to the 5′ and 3′-UTRs, five elements in FHV RNA 1 are required for replication: a *cis*-acting Response Element (*cis*-RE) at nt 68–205; the Proximal/Distal Subgenomic Control Elements (PSCE/DSCE) at nt 1229–1239 and nt 2282–2777; an internal response element (intRE) at nt 2322–2501; and a 3′ RE at nt 2735–3011. Similarly, in addition to the 5′ and 3′-UTRs, nt 186–217 in FHV RNA 2 form a 5′ Stem-Loop (5′ SL) required for RNA 2 encapsidation, and an intRE at nt 536–616 is required for RNA 2 replication. All of these previously characterized elements are conserved in our analysis.

Some of the recombination events detected in our analysis correspond to those previously observed to be present in FHV-defective genomes (14). However, deep-sequencing reveals a far richer distribution or quasi-species of defective genomes than has previously been observed using standard molecular cloning and polymerase chain reaction techniques. To see which regions of the FHV genome were retained during passaging, we plotted the frequency with which every nucleotide was excised among all of the detected recombinant RNAs. This reveals conserved regions of the viral genome. As can be seen in Figure 4, the two genomic RNAs show conservation of the 5′ and 3′-UTRs and some short internal motifs. There are three conserved regions in FHV RNA 2: nt 1–248, nt 520–725 and nt 1235–1400; and three conserved regions in FHV RNA 1: nt 1–300, nt 1105–1248 and nt 2308–3107. These observations correlate well with previous studies that have demonstrated the necessity for the 5′ and 3′-UTRs for efficient RNA replication (31–35). Similarly, a short motif is present in RNA 2 at nt 186–217 that forms a bulged stem loop and acts as a signal for RNA 2 packaging into virions (36). We would observe conservation of this packaging motif, as we only sequenced encapsidated RNAs. The internal regions at nt 538–616 in RNA 2 and at nt 68–205, nt 1227–1243 and nt 2322–3011 in RNA 1 have also previously been demonstrated to contain sequence motifs that are required for replication (31,33,37). The fact that we find all of these previously described motifs to be so well conserved in our analysis confirms their necessity for replication and packaging. This simple analysis demonstrates how regions of functional importance can be discovered through the use of NGS data without any prior knowledge of the lifecycle or genomic structure of a virus.

## DISCUSSION

There are number of highly cited software packages already available that address the complex issue of detecting recombination junctions. In contrast to these packages that extract individual segments of a pre-determined length and position from a read, ViReMa provides a unique approach by dynamically generating moving segments for alignment. After an initial seed-based alignment, a new read segment is obtained from any unaligned nucleotides at the 3′ end of the read. Alternatively, if a mapping cannot be found, the seed position is adjusted by trimming a single nucleotide from the beginning of the current segment. Consequently, ViReMa provides a highly sensitive and versatile platform for recombination discovery and it is not limited to specific reads lengths and it can handle reference genomes of any size. ViReMa is also capable of detecting multiple recombination events within a read, insertions and substitutions as well as more complex recombination events where they may be a small number of inserted nucleotides between recombination junctions.

Our algorithm is aimed at detecting the maximum number of recombination events and is almost exhaustive in its search for recombination events. In our analysis of over 31 million reads, >2.35 million recombination events were detected, 180 075 recombination events could not be unambiguously identified and <11 000 reads could not be mapped. Owing to artifactual recombination events that inevitably creep into cDNA-sequencing libraries (38), such an exhaustive search prompts consideration of the handling of false-positive recombination events. Many recombination detection algorithms are focused on the detection of splice-junctions in eukaryotic mRNA or in the detection of chromosomal rearrangements or fusions in the DNA of tumorigenic cells. These programs implement strict noise-reducing filters to remove any potential false-positive hits and any putative junction must be confirmed by the presence of multiple aligning reads or by paired reads that span a recombination junction. This is suitable in cases such as eukaryotic RNA splicing, as there should be only one or a limited number of biologically correct fusion junctions and as these junctions are likely to contain canonical splicing site consensus sequences.

However, this is not a good assumption in the case of RNA or DNA recombination in viral genomes. As many thousands of genome copies may be generated in a single replication cycle and as a deeply-sequenced viral genome will contain reads derived from a large pool of viral quasi-species, we should expect to find a wide range of possible recombination sites. Moreover, the mechanisms of DNA or RNA recombination may vary between species. For example, RNA template switching has been shown to occur most frequently in AU-rich regions of the brome mosaic virus RNA genome (39), whereas poliovirus has been demonstrated to favor GC-rich tracts (9). Consequently, we cannot exclude events based on the sequence information alone. Therefore, suitable controls must be used to obtain a base-line rate for artifactual recombination during the generation of the cDNA-sequencing libraries. This can be achieved by comparing the recombination in the viral genome with non-viral templates such as *in vitro* transcribed RNA. Similarly, artifactual recombination can be directly detected by mixing separate samples before cDNA library generation (9).

Our analysis of FHV demonstrates that by isolating a small number of virus particles, deep sequencing the encapsidated RNA and mapping the positions of recombination events, functional RNA motifs can be discovered. In principle, the approach laid out here would be possible even without knowledge of the genome sequence of the virus, as this can be assembled *de novo* from the sequence dataset (3). Also, the sample would not need to be purified as with sufficient sequencing read depth, the non-viral sequence reads can be removed computationally to reveal just the relevant virus data (40). We are constantly facing the threat of emerging pathogens, as exemplified by recent coronavirus and influenza virus outbreaks. It is therefore critical to develop methodology that allows researchers to quickly identify and characterize important features of a viral genome while only having available limited knowledge or means to study an outbreak virus.

## FUNDING

Supported by a European Molecular Biology Organization Long-Term Fellowship (to A.R.). Funding for open access charge: The National Institutes of Health [R37-GM034220 to J.E.J.].

*Conflict of interest statement*. None declared.
